# Recovery kinetics of short-term depression of GABAergic and glutamatergic synapses at layer 2/3 pyramidal cells in the mouse barrel cortex

**DOI:** 10.3389/fncel.2023.1254776

**Published:** 2023-09-25

**Authors:** Aniello Lombardi, Qiang Wang, Maik C. Stüttgen, Thomas Mittmann, Heiko J. Luhmann, Werner Kilb

**Affiliations:** ^1^Institute of Physiology, University Medical Center of the Johannes Gutenberg University, Mainz, Germany; ^2^Institute of Pathophysiology, University Medical Center of the Johannes Gutenberg University, Mainz, Germany

**Keywords:** barrel cortex, synaptic plasticity, pyramidal neurons, PV-interneurons, patch clamp, optogenetics

## Abstract

**Introduction:**

Short-term synaptic plasticity (STP) is a widespread mechanism underlying activity-dependent modifications of cortical networks.

**Methods:**

To investigate how STP influences excitatory and inhibitory synapses in layer 2/3 of mouse barrel cortex, we combined whole-cell patch-clamp recordings from visually identified pyramidal neurons (PyrN) and parvalbumin-positive interneurons (PV-IN) of cortical layer 2/3 in acute slices with electrical stimulation of afferent fibers in layer 4 and optogenetic activation of PV-IN.

**Results:**

These experiments revealed that electrical burst stimulation (10 pulses at 10 Hz) of layer 4 afferents to layer 2/3 neurons induced comparable short-term depression (STD) of glutamatergic postsynaptic currents (PSCs) in PyrN and in PV-IN, while disynaptic GABAergic PSCs in PyrN showed a stronger depression. Burst-induced depression of glutamatergic PSCs decayed within <4 s, while the decay of GABAergic PSCs required >11 s. Optogenetically-induced GABAergic PSCs in PyrN also demonstrated STD after burst stimulation, with a decay of >11 s. Excitatory postsynaptic potentials (EPSPs) in PyrN were unaffected after electrical burst stimulation, while a selective optogenetic STD of GABAergic synapses caused a transient increase of electrically evoked EPSPs in PyrN.

**Discussion:**

In summary, these results demonstrate substantial short-term plasticity at all synapses investigated and suggest that the prominent STD observed in GABAergic synapses can moderate the functional efficacy of glutamatergic STD after repetitive synaptic stimulations. This mechanism may contribute to a reliable information flow toward the integrative layer 2/3 for complex time-varying sensory stimuli.

## 1. Introduction

Extracting valid information from the complex and non-stationary natural environment requires adaptation of sensory systems, covering temporal domains from ms periods in sensory processing to hours or years for persistent memories (for review Wark et al., 2007). One mechanism for such an adaptation is short-term plasticity (STP), which encompasses reversible, activity-dependent alterations in synaptic transmission lasting between hundreds of milliseconds to several seconds (for review Regehr, 2012; Jackman and Regehr, 2017). Different temporal trajectories of STP influence transmission properties in sensory processing, establishing, e.g., adaptive gain control or temporal filtering (Fortune and Rose, 2001; Anwar et al., 2017; Motanis et al., 2018). However, it is less understood how different dynamics of STP decay at excitatory and inhibitory synapses contribute to sensory processing (Chapman et al., 2022).

The barrel cortex is, due to its precise columnar organization an established experimental paradigm for sensory information processing (for review Schubert et al., 2007; Feldmeyer et al., 2013; Stüttgen and Schwarz, 2018; Staiger and Petersen, 2021). A cortical barrel receives sensory information from a single mystacial whisker through topologically organized functional connections via the trigeminal nucleus and the ventral posterio-medial nucleus of the thalamus (for extralemniscal pathways see Feldmeyer et al., 2013; Luhmann, 2023). The whisker-related information of this lemniscal pathway is relayed mainly to spiny stellate cells in layer 4 (L4), which project mostly to neurons in L2/3 within the same barrel column. L4 to L2/3 connections are mediated by AMPA and NMDA receptors with high release probability and low synaptic efficacy (Feldmeyer et al., 2002; Silver et al., 2003; Lefort et al., 2009). In addition to these excitatory connections, inhibitory GABAergic interneurons (IN) are also essential elements of the circuit in the barrel cortex (Reyes-Puerta et al., 2015a; Yang et al., 2017; Baruchin et al., 2022). L2/3 IN represent about 11% of neurons in L2/3 (Lefort et al., 2009; Feldmeyer et al., 2018) and receive synaptic inputs from L4 spiny stellate cells with more efficient synapses than L2/3 pyramidal neurons (PyrN), thereby promoting substantial feedforward inhibition of PyrN (Helmstaedter et al., 2008). In addition, L2/3 fast-spiking IN receive both excitatory and inhibitory inputs from L2/3 neurons (Xu and Callaway, 2009; Pala and Petersen, 2015), thereby modifying the integration of sensory signals in this layer (Feldmeyer et al., 2013, 2018). The synaptic targets and properties depend on the subpopulation of neocortical GABAergic IN (see Ascoli et al., 2008; Tremblay et al., 2016 for a detailed classification of neocortical interneurons). In L2/3 only ca. 25% of the GABAergic interneurons express parvalbumin (PV) (Lee et al., 2010; Xu et al., 2010; Rudy et al., 2011). Baskets cells, which represent a major fraction of PV-IN, show less barrel-restricted axonal projections in L2/3 than L4 basket cells, making them suitable elements to mediate intra-and intercolumnar inhibition (Helmstaedter et al., 2009a,b).

Information transfer within this system cannot be understood without considering the plasticity of its synaptic connections (Gutnisky et al., 2017; Feldmeyer et al., 2018). Thalamocortical excitatory inputs to L4 (Beierlein et al., 2003; Cruikshank et al., 2010) as well as connections from L4 to L2/3 PyrN and L2/3 IN (Feldmeyer et al., 2002; Helmstaedter et al., 2008) display a pronounced short-term depression (STD), which can affect intracortical information transfer (Pinto et al., 2000; Gutnisky et al., 2017; Katz and Lampl, 2021). To understand the role of STD on cortical information processing, it is necessary to know the time span of STD in both excitatory and inhibitory synapses. However, to our knowledge only three studies analyzed the duration of STD in cortical neurons and reported that the STD of excitatory postsynaptic potentials (EPSPs) lasts between 700 ms and 8 s (Chung et al., 2002; Cohen-Kashi Malina et al., 2013; Li and Glickfeld, 2023). To resolve at which level this transient adaptation to repetitive sensory stimulation occurs and to understand its impact on sensory processing, an analysis of the kinetic properties of STD for EPSCs and IPSCs is necessary.

In order to investigate the kinetics of STP for inhibitory and excitatory synapses and to reveal how these kinetic properties affect synaptic integration, we performed whole-cell recordings from identified L2/3 PyrN and IN of the barrel cortex and analyzed STP induced either by electrical stimulation of afferents in L4 or optogenetic burst stimulation of parvalbumin-positive GABAergic INs. Our results demonstrate a prominent STD of glutamatergic and GABAergic synapses, with slower decay kinetics at GABAergic synapses. The simultaneous STD of both inputs resulted in mostly unaffected EPSPs in L2/3 PyrN after electrical burst stimulation.

## 2. Methods

### 2.1. Animals

All animal experiments were approved by the local German ethics committee (Landesuntersuchungsamt Rheinland-Pfalz, Koblenz, Germany; Az. 23177-07/G19-1-085) and were performed according to European and German laws (European Communities Council Directive, 86/609/ECC). Mice were kept in groups of 2–3 animals per cage at 12/12 h day/night cycles and had *ad libitum* access to food and water. Experiments were performed in coronal cortical slices of 24 adult parvalbumin Cre (PV-Cre) (B6; 129P2-Pvalbtm1(cre)Arbr/J; The Jackson Laboratory, Bar Harbor, Maine, USA, JAX #017320) knock-in mice at postnatal day (P) 30 – P60. In 11 of these animals, unilateral viral injections of a solution containing 3.6 × 10^12^ mL^−1^ rAAV2/EF1a-DIO-ChR2(H134R)-eYFP were performed at P15-20 under deep isoflurane anesthesia. Some experiments were performed in parvalbumin reporter animals to enable patch-clamp experiments from visually identified fluorescent PV-IN. For this purpose, PV-Cre mice were crossbred with a tdTomato reporter mouse line (B6.Cg-Gt (ROSA)26Sortm14(CAG-tdTomato)Hze/J, The Jackson Laboratory, JAX #007914), and four heterozygous mice from the offspring were used for patch-clamp experiments from PV-IN visually identified by their tdTomato fluorescence. Few experiments for the pharmacological characterization of stimulus evoked IPSCs were performed in one C57Bl/6N mouse.

### 2.2. Solutions and drugs

The artificial cerebrospinal fluid (ACSF) consisted of (in mM) 125 NaCl, 25 NaHCO_3_, 1.25 NaH_2_PO_4_, 1 MgCl_2_, 2 CaCl_2_, 2.5 KCl, 10 glucose and was equilibrated with 95% O_2_ / 5% CO_2_ at least 1 h before use (pH 7.4, osmolarity 306 mOsm). The slices for the PV-reporter mice were cut in a choline chloride-based solution consisting of (in mM) 37.5 (choline chloride, 87 NaCl, 2.5 KCl, 1.25 NaH_2_PO_4_, 0.5 CaCl_2_, 7 MgCl_2_, 25 NaHCO_3_ and 25 d-glucose). The pipette solution was composed of (in mM) 128 K-gluconate, 2 KCl, 4 NaCl, 1 CaCl_2_, 11 EGTA, 10 K-HEPES, 2 Mg_2_-ATP, 0.5 Na-GTP and 2 lidocaine-N-ethyl chloride (pH adjusted to 7.4 with KOH and osmolarity to 306 mOsm with sucrose). Dimethylsulfoxide (DMSO) and lidocaine-N-ethyl chloride were obtained from Sigma-Aldrich (St. Louis, Missouri); gabazine (SR95531), DL-2-Amino-5-phosphonopentanoic acid (APV) and 6-Cyano-7-nitroquinoxaline-2,3-dione (CNQX) were obtained from Biotrend (Cologne, Germany). APV, CNQX and gabazine were used from stock solutions in DMSO. The DMSO concentration of the final solution never exceeded 0.1%.

### 2.3. Brain slice preparation

For slice preparation, P30-P60 animals were deeply anesthetized with enflurane (Ethrane, Abbot Laboratories, Wiesbaden, Germany) or isoflurane (Piramal Critical Care, Hallbergmoos, Germany) and decapitated. The brain was quickly isolated and transferred to ice-cold artificial cerebrospinal fluid (ACSF) equilibrated with 95% O_2_ / 5% CO_2_, and allowed to recover for at least 2 min. The isolated brain was fixed on the stage of a vibratome and coronal slices (300 μm for the PV reporter mice and 400 μm for all other mice) containing the somatosensory cortex were cut using a vibratome (VT1200 S, Leica, Germany). Slices from PV-reporter animals were cut (at <4°) and subsequently incubated in choline chloride based cutting solution for 20 min at 37°C before they were transferred to standard ACSF. The other slices were cut in standard ACSF at <4°C and afterward directly transferred into standard ACSF at room temperature. Slices were allowed to recover for at least 1 h after the cutting procedure.

### 2.4. Whole-cell patch-clamp recordings

Single cortical slices were placed in a submerged-type recording chamber mounted on the stage of an upright microscope (BX-51W, Olympus, Japan) equipped with a differential interference-contrast (DIC) optics and a set of fluorescence filters to identify tdTomato fluorescence. Slices were continuously perfused with oxygenated standard ASCF at 31–35°C. Whole-cell patch clamp recordings were performed from visually identified neurons in L2/3, PyrN were identified by their appearance in the DIC image and PV-IN by the tdTomato fluorescence signal. The patch pipettes were pulled from borosilicate glass (GB200F-8, Science Products, Hofheim am Taunus, Germany) and had a resistance of 3–5 MΩ when filled with the pipette solution. The whole-cell patch-clamp recordings were performed with either an EPC10 amplifier controlled by TIDA software (Heka electronics, Lambrecht, Germany) or an Axopatch-200B amplifier connected to Digidata 1440A and pClamp 11.1 software (Molecular Devices, US). Synaptic stimulations were provided by a stimulus isolator (A360, World Precision Instruments, US) delivering electric pulses of 30–400 μA in amplitude and 50 μs in pulse duration through a low resistance borosilicate glass pipette (GB 150F-8P; Science Products, Germany) filled with normal ACSF. Stimulation pipettes were placed in L4 within the same barrel column as the recorded neuron to stimulate the projections from L4 to L2/3 PyrN and L2/3 IN (House et al., 2011).

### 2.5. Optogenetic stimulation

For optogenetic stimulation of the GABAergic inputs to L2/3 PyrN, the light sensitive cation channel Channelrhodopsin 2 (ChR2) was expressed in PV-IN using rAAV2/EF1a-DIO-ChR2(H134R)-eYFP viruses. The pAAV-Ef1a-DIO hChR2(E123A)-EYFP plasmid (addgene plasmid # 35507) was a gift from Karl Deisseroth. For virus injections, P15-20 PV-Cre mice were deeply anesthetized with isoflurane (3–5% for the initial induction and 1–2% during the whole surgical procedure), placed on a warming pad at 37°C, and fixed in a stereotactic frame (Kopf Instruments, TUJUNGA, CA). A small cranial opening was prepared at the level of the somatosensory cortex (AP: −2 mm, ML: 3.5 mm) under a dissecting microscope using a dental drill (Ultimate XL-F, NSK, Trier, Germany) and a small injection needle. Ca. 300 nL of viral solution containing 3.6 × 10^12^ mL^−1^ rAAV2/EF1a-DIO-ChR2(H134R)-eYFP viruses was slowly injected (150–300 nL/min) via a fine glass micropipette (Hirschmann Laborgeräte, Eberstadt, Germany) inserted ca. 300–600 μm below the pial surface. The functional experiments were performed in slices prepared from P30-P60 animals and therefore in all cases >14 days post injection. Optogenetic stimulation of ChR2-expressing PV-In was performed in a standard patch-clamp setup equipped with a 50 mW solid-state laser at 488 nm wavelength (Sapphire, Coherent, Dieburg, Germany). The laser beam was coupled to a 200 μm multimode fiber with a numerical aperture of 0.39 (Thorlabs, Munich, Germany) via a collimator (Schäfer & Kirchhoff, Hamburg, Germany). Laser illumination was controlled by a mechanical shutter (Uniblitz, Rochester, USA) connected to the digital output of the EPC10. The fiber was fixed in a glass capillary and positioned via a standard micromanipulator (Luigs & Neumann, Ratingen, Germany) directly (<100 μm) above the cortical slice at an angle of ca. 25°. The estimated spot dimension was ca. 290 μm × 690 μm resulting in a maximal power density of ca. 37 mW/mm^2^ (with a maximal output power at the end of the fiber of 23.8 mW). For repetitive stimulation a pulse duration of 1 ms at 30% of maximal laser power were used. Control experiments revealed that the PV-INs can reliably follow these stimuli with single action potentials.

### 2.6. Stimulation and analysis

Excitatory and inhibitory postsynaptic currents (EPSCs and IPSCs, respectively) were evoked at stimulation intensities between 30 and 400 μA, adjusted individually for each cell to achieve reliable postsynaptic responses. Stimulation intensities were identical for control, burst and test pulses. EPSCs and IPSCs were isolated in voltage-clamp mode by clamping the membrane potential at −60 mV (estimated reversal potential of GABAergic synapses) or 0 mV (estimated reversal potential of glutamate receptors). In a few experiments, 10 μM gabazine or a mixture of 10 mM CNQX +30 μM APV were used to verify the unequivocal identification of IPSCs and EPSCs by different holding potentials. The latency of EPSCs and IPSCs was determined between the onset of the electrical/laser stimulus and the onset of the synaptic response. Amplitudes of EPSCs and IPSCs were determined from the baseline current measured directly before stimulation. The rise time of the EPSCs and IPSCs was determined at 20–80% levels, and the decay time constant was defined as the latency between peak and 37% of the peak amplitude. The synaptic conductance was estimated by dividing the EPSC/IPSC amplitude with the estimated electromotive force for glutamatergic and GABAergic responses, respectively. Current clamp experiments were performed at a standard potential of ca. −60 mV. Peak amplitudes of excitatory postsynaptic potentials (EPSPs) were determined from the membrane potential measured directly before stimulation. For quantification, amplitudes of EPSCs, IPSCs and EPSPs obtained at defined latencies after the end of the burst were related to the amplitudes of test stimuli delivered 10 s before the onset of the burst stimulation.

### 2.7. Statistics

Data are presented as mean ± SEM. Statistical analyses were performed using Systat11 (Systat Software, Point Richmond, CA). Control and test pulses at the different latencies were compared pairwise with the Wilcoxon signed-rank test. Comparisons between groups were performed with the Kruskal-Wallis One-Way test. *P*-values of multiple tests were corrected by the Bonferroni-Holmes method. Statistical significance was assigned at *p < 0.05, **p < 0.01, and ***p < 0.001. Box plots were generated with Systat11 and represent lower quartile, median, and upper quartile with whiskers indicating minimum/maximum of data points within the 1.5-fold interquartile range.

## 3. Results

### 3.1. Properties of glutamatergic and GABAergic synaptic inputs

**Figure 1:**
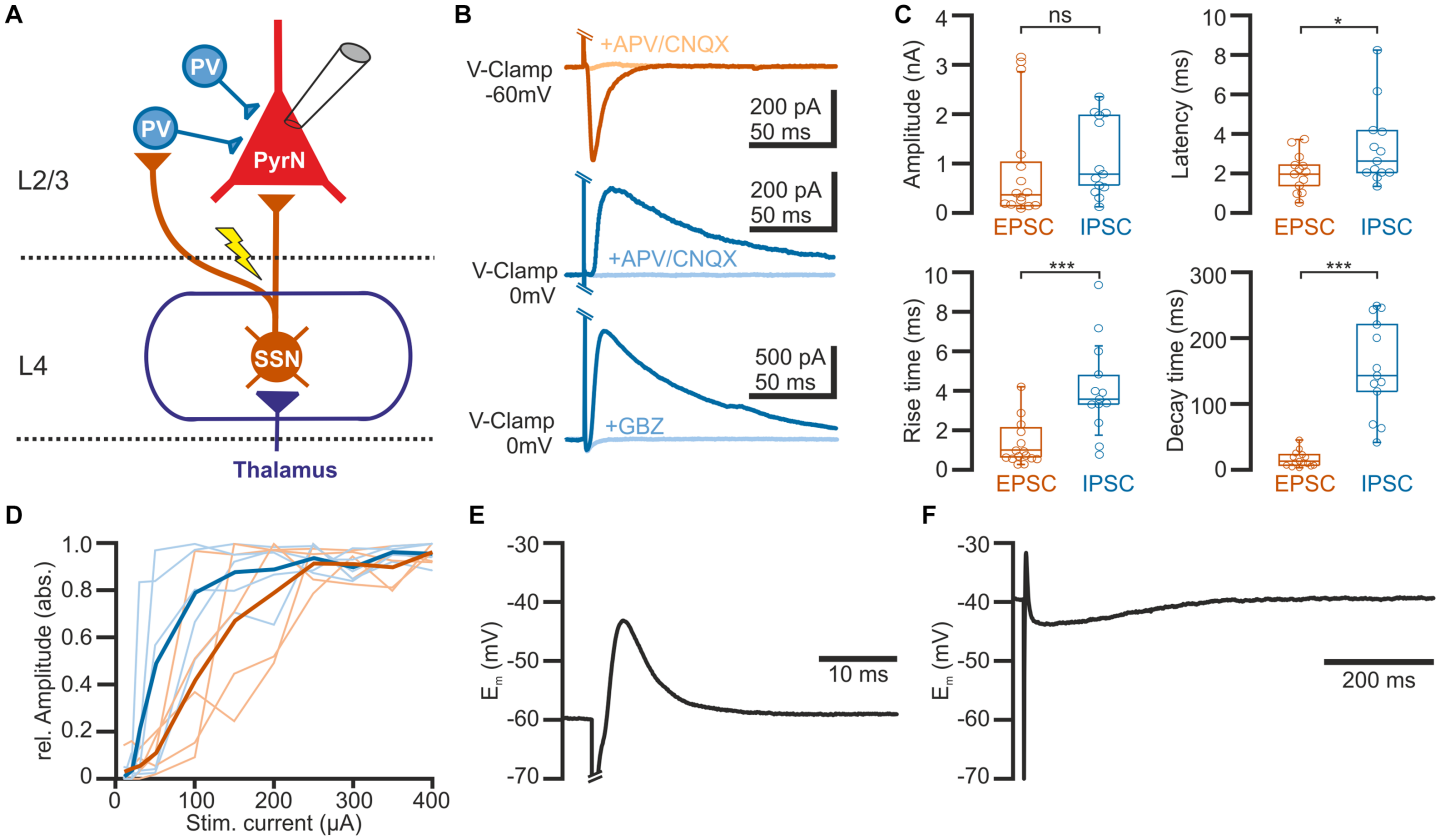
Properties of synaptic connections between L4 and L2/3 pyramidal neurons. **(A)** Schematic representation of the experimental approach and some basal circuitry elements in the barrel cortex. The yellow arrow indicates the site of electric stimulation in L4, activating projections from spiny stellate neurons (SSN) toward both parvalbumin-interneurons (PV-IN) and pyramidal neurons (PN) in L2/3. **(B)** Typical current responses of a L2/3 pyramidal neuron (PN) upon suprathreshold electrical stimulation. The upper traces are recorded at a holding potential of-60 mV to isolate the glutamatergic component (EPSC). The middle and lower traces are recorded at 0 mV to isolate the GABAergic component (IPSC). Note that EPSCs are inhibited by the glutamatergic antagonists CNQX (10 μM) + APV (30 μM) and IPSCs by either CNQX (10 μM) + APV (30 μM) (middle traces) or the GABAergic antagonist gabazine (10 μM, lower traces). **(C)** Boxplots illustrating the properties of the EPSCs and IPSCs. **(D)** Input–output curve for EPSCs (red) and IPSCs (blue). The light traces represent responses from individual neurons and the bold traces depict mean values. **(E)** Typical voltage response of a L2/3 PyrN from a standard potential of −60 mV, illustrating the fast excitatory postsynaptic potential (EPSP), sculptured by the kinetics of the EPSC and the IPSC. **(F)** The voltage response recorded from a standard potential of −40 mV reveals the underlying GABAergic component of the response. Note the longer time scale in this plot. Significance is indicated by *p < 0.05 and ***p < 0.001.

In order to investigate the short-term plasticity (STP) of synaptic connections in barrel cortex, we used an electric stimulus delivered by a monopolar electrode located in L4 and recorded the postsynaptic responses in L2/3 pyramidal neurons in the stimulated barrel column (see Figure 1A). Voltage-clamp recordings at a holding potential (E_h_) of-60 mV, which is close to the estimated GABA reversal potential (E_GABA_) and thus minimizes contamination with GABAergic synaptic inputs, revealed that a single electrical stimulus induced an excitatory postsynaptic current (EPSC) with a maximal amplitude of 0.9 ± 0.3 nA (*n* = 15 cells, *n* = 9 animals, Figures 1B,C), corresponding to a conductance of 18.7 ± 3.5 pS (*n* = 15). The EPSCs were blocked by 97.6 ± 0.7% (*n* = 6 cells, *n* = 4 animals) in the presence of 10 μM CNQX/30 μM APV. This EPSC occurred with a latency of 2 ± 0.2 ms, had a rise time of 1.4 ± 0.3 ms and a decay time constant of 19.4 ± 3.1 ms, which is in line with the properties of a monosynaptic glutamatergic EPSP in this L4 to L2/3 connection (Feldmeyer et al., 2002).

Inhibitory postsynaptic currents (IPSCs) upon a single electrical stimulus were recorded at 0 mV to minimize the contamination by glutamatergic EPSCs (Figures 1B,C). These stimulus-dependent IPSCs (*n* = 13 cells, *n* = 8 animals) had a maximal amplitude of 1.1 ± 0.2 nA, corresponding to a conductance of 15.1 ± 4.8 pS (*n* = 13). Compared to the EPSCs, the IPSCs had a significantly (*p* = 0.0007) longer rise time of 4.9 ± 0.8 ms, a significantly (*p* < 0.0001) longer decay time constant of 164.5 ± 2.9 ms, and occurred with a significantly (*p* = 0.04) longer latency of 3.3 ± 0.8 ms. The IPSCs were blocked by 88.5 ± 4.6% (*n* = 8 cells, *n* = 3 animals) in the presence of 10 μM CNQX/30 μM APV and by 96.6 ± 1.5% (*n* = 5 cells, *n* = 3 animals) in the presence of 10 μM gabazine (Figure 1B). This pharmacology, the longer latency and the slower kinetic properties indicate that these IPSCs represent mainly disynaptic stimulus-evoked responses, as expected by the canonical circuit of the barrel cortex (see Figure 1A; Feldmeyer et al., 2013). Both, IPSCs and EPSCs showed a steep dependency on stimulation strength, with the IPSC amplitude saturating at lower stimulus intensities (Figure 1D).

Under current-clamp conditions at a standard potential of −60 mV (i.e., when E_m_ is close to E_GABA_), the single electrical stimulus induced a postsynaptic response consisting of a fast depolarizing membrane deflection (Figure 1E). This excitatory postsynaptic potential (EPSP) occurred at a latency of 2.1 ± 0.1 ms (*n* = 18 cells, *n* = 8 animals), had a mean amplitude of 11.3 ± 2.1 mV, a rise time of 0.6 ± 0.1 ms, and a duration of 8.8 ± 2.7 ms. Under conditions when the standard membrane potential was positive to E_GABA_, this EPSP was followed by a longer hyperpolarizing component (Figure 1F), indicating that the shape of the voltage response was mediated by both glutamatergic and GABAergic synaptic inputs.

### 3.2. Glutamatergic and GABAergic synaptic inputs in layer 2/3 pyramidal neurons exhibit short-term depression

**Figure 2:**
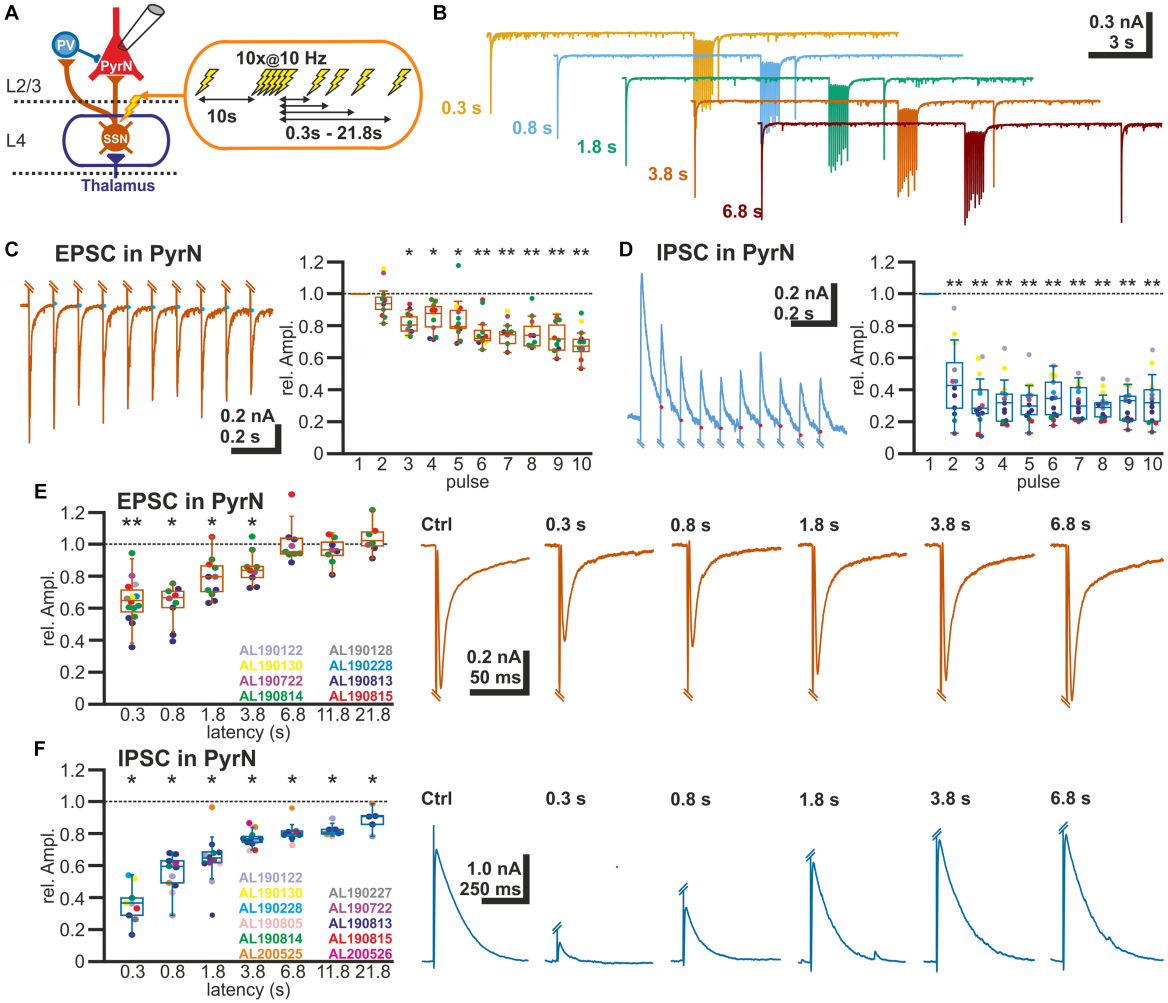
Short-term plasticity of glutamatergic and GABAergic synapses at L2/3 pyramidal neurons. **(A)** Schematic representation of recording and stimulation condition. An electrical stimulation protocol consisting of a control stimulus, followed by a burst stimulation and test stimuli applied at latencies between 0.3 and 21.8 s after the burst, was applied via a monopolar electrode located in L4. **(B)** Typical current responses to the stimulation protocol at five different latencies. **(C)** Amplitude of EPSCs during the burst stimulation. The blue dots indicate the basis for amplitude detection based on the exponential decay of the preceding EPSC. The box plot illustrates the significant reduction in the EPSC amplitude during the burst. Color code for data points as indicated in panel **(E)**. **(D)** Amplitude of IPSCs during the burst stimulation. The red dots indicate the basis for amplitude detection based on the exponential decay of the preceding IPSC. The box plot illustrates the significant reduction in the EPSC amplitude during the burst. Color code for data points as indicated in panel **(F)**. **(E)** Boxplot illustrating that the relative amplitude of EPSCs (determined at −60 mV) reveal significant STD lasting ca. 1.8 s. In the right panels, typical current traces are displayed. **(F)** Relative amplitude of IPSCs (determined at −0 mV) reveal a significant STD lasting at least 21.8 s. In the right panels typical current traces are displayed. Significance in panel **(C,D)** is tested by ANOVA with Tuckey *post-hoc* test and in panel **(E,F)** with a Wilcoxon test; significance is indicated by *p < 0.05 and **p < 0.01.

To analyze the STP at excitatory and inhibitory synapses in L2/3, we applied via a monopolar stimulation electrode positioned in L4 a stimulation sequence consisting of a control stimulus, followed after 10 s by a burst of 10 electrical pulses at a frequency of 10 Hz, and a subsequent test pulse given at latencies between 0.3 and 21.8 s after the end of the burst stimulation (Figures 2A,B). The amplitude of the test EPSC was related to the control EPSC for each test sequence. Burst stimulation induced a decline in the amplitude of the EPSCs evoked during the burst (Figure 2C). The last EPSC of the burst was reduced by 31.2 ± 3.3% (*n* = 13 cells, *n* = 8 animals). An even stronger decline in the amplitudes was observed for the IPSCs (Figure 2D). The last IPSC of the burst was reduced by 66.4 ± 4.1% (*n* = 13 cells, *n* = 8 animals).

The reduced amplitude of the EPSCs persisted to the test-pulse applied at short latencies after the burst (Figures 2B,E). The mean EPSC amplitude was significantly (*p* < 0.05, Wilcoxon paired test) reduced by 35.2% ± 3.5% (*n* = 15 cells, *n* = 8 animals) for a pulse given 300 ms after the last stimulus of the burst, by 37.6% ± 4.2% (*n* = 9 cells, *n* = 4 animals) for 0.8 s latency, by 20.9% ± 3.8% (*n* = 11 cells, *n* = 4 animals) for 1.8 latency, and by 14.8% ± 3.5% (*n* = 9 cells, *n* = 4 animals) for 3.8 s latency (Figure 2E; Table 1).

**Table 1:** Amplitudes of EPSCs, IPSCs or EPSPs at different postburst latencies for different experimental conditions.

**Relative amplitude of electrical induced EPSCs in Ly 2/3 pyramidal neurons**							
**latency**	**0.3 s**	**0.8 s**	**1.8 s**	**3.8 s**	**6.8 s**	**11.8 s**	**21.8 s**
Mean	64.8%	62.4%	79.1%	85.2%	100.9%	96.3%	104.1%
S.E.M.	3.5%	4.2%	3.8%	3.5%	4.2%	3.0%	3.7%
*n*	15	9	11	9	9	8	7
*p*	0.0046	0.0384	0.0266	0.0434	0.2604	0.4152	0.3846
**Relative amplitude of electrical induced IPSCs in Ly 2/3 pyramidal neurons**							
**latency**	**0.3 s**	**0.8 s**	**1.8 s**	**3.8 s**	**6.8 s**	**11.8 s**	**21.8 s**
Mean	35.9%	50.4%	59.0%	70.5%	72.1%	70.5%	74.3%
S.E.M.	4.0%	5.6%	6.4%	6.6%	9.3%	11.8%	15.1%
*n*	9	12	13	12	9	7	6
*p*	0.0307	0.0167	0.0201	0.0234	0.0352	0.0180	0.0359
**Relative amplitude of initial component of electrical induced EPSCs in Ly 2/3 PV-interneurons**							
**Latency**	**0.3 s**	**0.8 s**	**1.8 s**	**3.8 s**	**6.8 s**	**11.8 s**	**21.8 s**
Mean	62.2%	67.9%	71.7%	70.0%	84.7%	103.2%	103.4%
S.E.M.	6.3%	5.3%	5.2%	8.2%	4.8%	5.1%	6.5%
*n*	10	10	10	8	10	10	10
*p*	0.0354	0.0304	0.0253	0.0352	0.0374	0.6465	1.0152
**Relative charge transfer in electrical induced multisynaptic EPSCs in Ly 2/3 PV-interneurons**							
**Latency**	**0.3 s**	**0.8 s**	**1.8 s**	**3.8 s**	**6.8 s**	**11.8 s**	**21.8 s**
Mean	61.8%	68.9%	74.8%	74.0%	83.4%	99.5%	97.3%
S.E.M.	5.7%	7.0%	4.3%	4.9%	4.9%	4.8%	3.0%
*n*	10	10	10	8	10	10	10
*p*	0.0354	0.0304	0.0253	0.0469	0.0853	1.7570	0.9594
**Relative amplitude of optogenetically induced IPSCs in Ly 2/3 pyramidal neurons**							
**Latency**	**0.3 s**	**0.8 s**	**1.8 s**	**3.8 s**	**6.8 s**	**11.8 s**	**21.8 s**
Mean	59.8%	65.1%	76.9%	84.3%	91.1%	91.0%	89.8%
S.E.M.	3.4%	2.3%	2.9%	2.4%	1.4%	2.6%	2.8%
*n*	10	10	10	10	10	10	9
*p*	0.0056	0.0112	0.0169	0.0225	0.0346	0.0415	0.0538
**Relative amplitude of EPSPs in Ly 2/3 pyramidal neurons**							
**Latency**	**0.3 s**	**0.8 s**	**1.8 s**	**3.8 s**	**6.8 s**	**11.8 s**	**21.8 s**
Mean	84.0%	97.8%	96.6%	108.3%	114.0%	103.2%	112.0%
S.E.M.	7.0%	4.5%	3.9%	5.6%	11.0%	5.2%	6.1%
*n*	11	11	10	10	10	10	10
*p*	0.9147	1	1	0.9594	1	1	1
**Relative amplitude of EPSPs in Ly 2/3 PN after ChR2 burst-like activation**							
**Latency**	**0.3 s**	**0.8 s**	**1.8 s**	**3.8 s**	**6.8 s**	**11.8 s**	**21.8 s**
Mean	117.4%	121.9%	103.1%	99.0%	90.4%	101.1%	100.1%
S.E.M.	5.1%	6.8%	3.5%	5.1%	5.8%	8.0%	6.7%
*n*	14	13	13	10	10	10	10
*p*	0.0232	0.0207	1	0.6079	0.0543	0.6752	0.9594

In contrast, the amplitude of the GABAergic IPSCs showed a longer lasting STD after burst-like electrical stimulation (Figure 2F). Here the IPSC amplitude was significantly reduced by 64.1% ± 4.0% (*n* = 9 cells, *n* = 7 animals) for 0.3 s latency, by 49.6% ± 5.6% (*n* = 12 cells, *n* = 6 animals) for 0.8 s latency, by 41% ± 6.4% (*n* = 13 cells, *n* = 7 animals) for 1.8 s latency, and by 29.5% ± 6.6% (*n* = 12 cells, *n* = 7 animals) for 3.8 s latency (Figure 2F; Table 1). Even 11.8 s after the burst stimulation the IPSC amplitude was still significantly (*p* = 0.018) reduced by 29.5% ± 11.8% (*n* = 7 cells, *n* = 6 animals).

In summary, these results indicate that both glutamatergic and GABAergic synaptic inputs toward L2/3 pyramidal neurons show a significant STD, but that this effect is more pronounced and showed a slower decay for the GABAergic inputs.

### 3.3. Glutamatergic synaptic inputs at layer 2/3 PV interneurons show short-term depression

**Figure 3:**
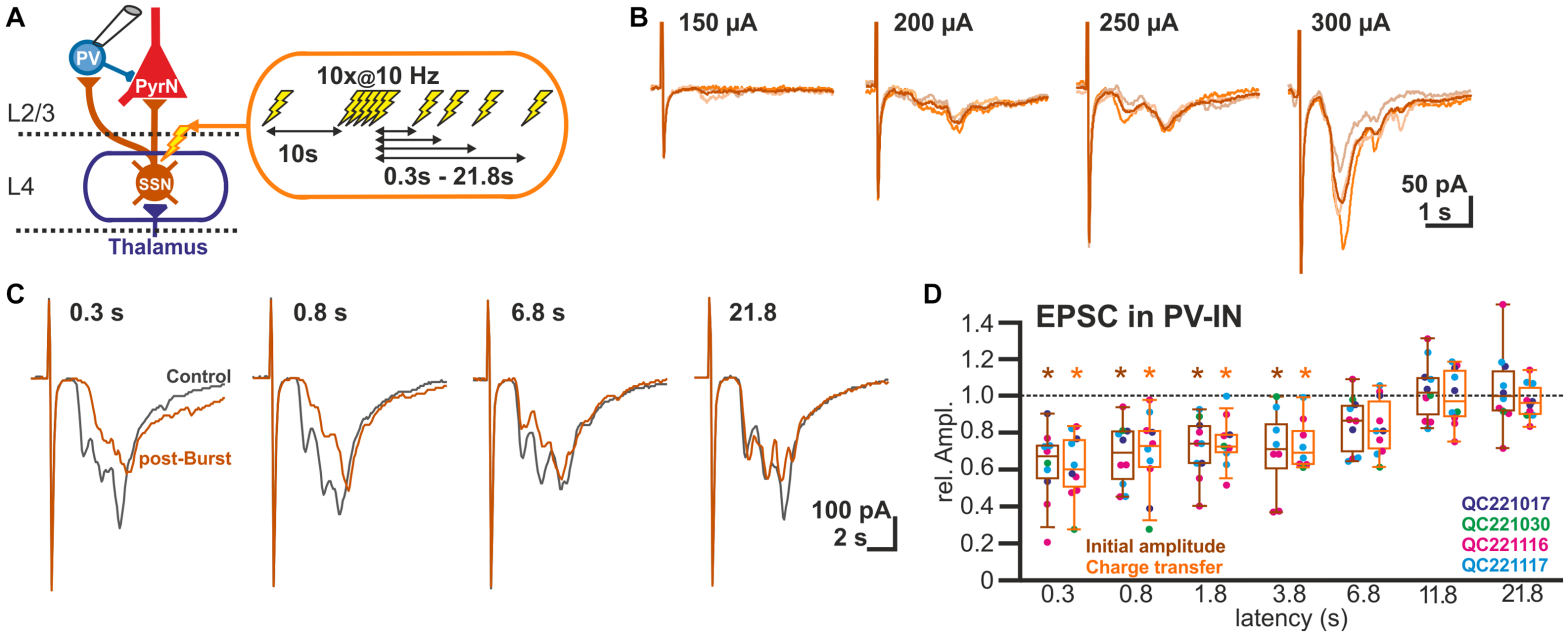
Short-term plasticity of glutamatergic synapses at L2/3 PV-IN. **(A)** Schematic representation of recording and stimulation condition used for the experiment shown in panel **(B)**. Each stimulation protocol consisted of a control stimulus, followed by a burst stimulation and test stimuli applied between 0.3 and 21.8 s after the burst. **(B)** Typical traces of glutamatergic EPSCs (recorded at −60 mV) upon increasing stimulus strength, illustrating that variable mono-and heterosynaptic inputs were observed in L2/3 PV-IN. Light traces represent single stimulations, dark traces the average of three consecutive stimulations. **(C)** Characteristic average traces obtained before (gray traces) and at given intervals after the burst stimulation (orange trances). The amplitudes of the responses were determined for the initial phase of the EPSCs. In addition, the charge transfer during the first 30 ms after the stimulus was determined. **(D)** Boxplot illustrating that initial amplitude (dark boxes) as well as charge transfer (light boxes) of glutamatergic EPSCs in PV-IN revealed a significant STD lasting 3.8 s. Significance is indicated by *p < 0.05.

As the GABAergic inputs to L2/3 PyrN likely represent disynaptic connections, mediated via glutamatergic afferents synapsing on fast-spiking IN (putative PV-positive basket cells; Wilent and Contreras, 2005), we next quantified STP of the glutamatergic synapses at L2/3 PV-IN (Figure 3A). For this purpose, we recorded EPSCs of L2/3 PV-IN, identified by their fluorescence in a PV-Cre transgenic animal, upon electrical burst stimulation of L4, using the same burst protocol as before. Electrical stimulation induced in these cells a series of mono-and heterosynaptic EPSCs (Figure 3B). As in our experiments it was not possible to reliably induce only a single monosynaptic EPSC, we analyzed the initial component of the excitatory response, which most probably reflects monosynaptic inputs. This initial EPSC component occurred 1.9 ± 0.1 ms (*n* = 10) after the electrical stimulus. It had an amplitude of 0.42 ± 0.07 nA (*n* = 10 cells, *n* = 4 animals), a rise time of 0.8 ± 0.1 ms and a decay time constant of 3.2 ± 0.3 ms. The burst stimulation induced a transient STD of the initial EPSC component (Figure 3C). It was significantly (*p* < 0.05, Wilcoxon paired test) reduced by 37.8% ± 6.3% (*n* = 10 cells, *n* = 4 animals) for a pulse given 0.3 s after the last stimulus of the burst, by 32.1% ± 5.3% (*n* = 10) for 0.8 s latency, by 28.3% ± 5.2% (*n* = 10) for 1.8 s latency, and by 30.0% ± 8.2% (*n* = 8) for 3.8 s latency (Figure 3D; Table 1). A comparable result was observed when STP of the polysynaptic glutamatergic response was quantified by analyzing the charge transfer during the first 30 ms after the response onset. The glutamatergic charge transfer was significantly (*p* < 0.05, Wilcoxon paired test) reduced by 38.2% ± 5.7% (*n* = 10 cells, *n* = 4 animals) for a pulse given 0.3 s after the last stimulus of the burst, by 31.1% ± 7.0% (*n* = 10) for 0.8 s latency, by 25.6% ± 4.3% (*n* = 10) for 1.8 s latency, and by 26% ± 4.9% (*n* = 12) for 3.8 s latency (Figure 3D; Table 1).

### 3.4. GABAergic synapses between PV interneurons and layer 2/3 pyramidal cells show short-term depression

**Figure 4:**
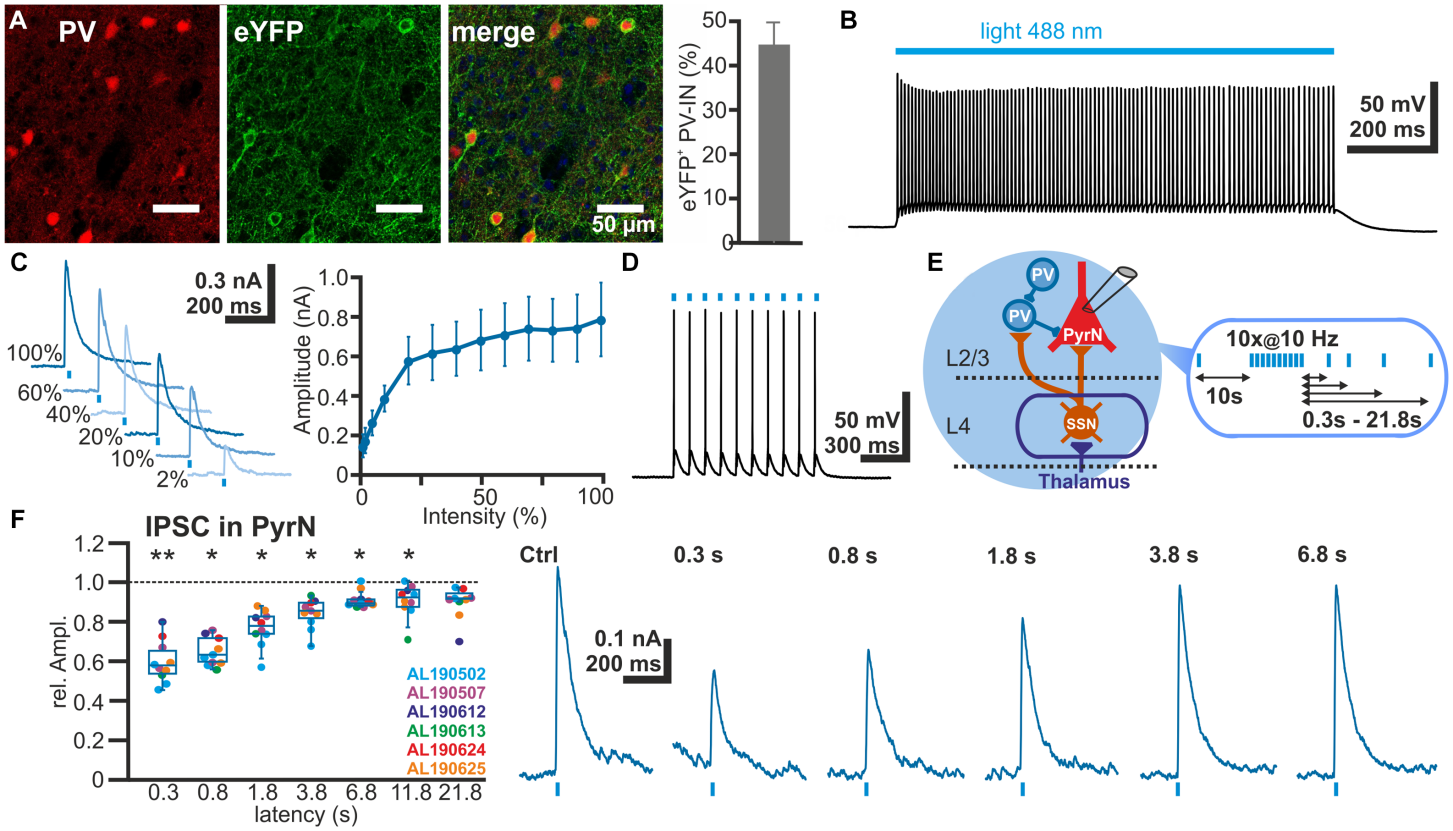
Short-term plasticity of GABAergic synapses between PV interneurons and L4 pyramidal neurons. **(A)** Immunhistochemical staining of PV (left image) and of ChR2-associated eYFP expression (middle image) in slices of transfected animals. Note the exclusive eYFP expression in PV^+^ neurons and that 44.7 ± 4.9% (*n* = 20 ROIs) of the PV^+^ interneurons are transfected (right bar diagram). **(B)** Illumination with a 488 nm light pulse induced a persistent depolarization in an eYFP^+^ neuron. **(C)** The light-induced GABAergic IPSCs (determined at 0 mV) in L2/3 PyrN depend on the laser intensity. Already at 2% of the maximal laser intensity, substantial inward currents were induced, which saturated at >30% laser intensity. **(D)** The light-induced neuronal activity in PV interneurons can reliably follow 10 Hz optogenetic stimulation. **(E)** Schematic representation of recording and stimulation condition used for the experiment shown in panel **(F)**. The stimulation protocol consisted of an optogenetic control stimulus, followed by optogenetic burst stimulation and optogenetic test stimuli applied between 0.3 and 21.8 s after the burst. GABAergic IPSCs were recorded in a L2/3 pyramidal neuron upon application of a stimulation protocol. **(F)** Boxplot illustrating that the relative amplitude of the optogenetically induced GABAergic IPSCs revealed a significant STD, lasting ca. 3.8 s. In the right panels typical current traces are displayed. Significance in panel **(F)** is indicated by *p < 0.05 and **p < 0.01.

To investigate the STP of GABAergic synapse between L2/3 PV-IN and L2/3 PyrN we used an optogenetic approach to selectively activate PV-IN. For this purpose, we expressed the light-gated cation channel ChR2 (Nagel et al., 2003) in PV-IN by intracranial injection of AAV containing a ChR2 construct (DIO-ChR2-eYFP) into a PV-cre mouse line. Clear eYFP expression was observed in 44.7 ± 4.9% (*n* = 20 ROIs in superficial layers, *n* = 5 slices, *n* = 2 animals) of all PV^+^ neurons (Figure 4A), indicating that a substantial fraction of the PV-IN could be activated by optogenetic stimulation. ChR2 was activated by laser illumination at a wavelength of 488 nm. The dimensions and spatial constrains of the laser stimulation (see section 2.5) implies that many PV-IN will be activated simultaneously with each laser pulse, although we cannot provide an exact number of the optogenetically stimulated PV-IN. Control recordings from ChR2-expressing neurons, as identified by their eYFP expression, revealed that laser illumination induced a membrane depolarization sufficient to induce spiking activity in five out of 11 eYFP^+^ PV-IN already at the lowest laser intensity tested (5%), while additional three PV-IN showed an AP at stimulation intensities of 10–30% (Figure 4B). In the remaining 3 PV-IN no APs are evoked even at the highest laser intensity. Characterization of the input–output relation demonstrated that already at 30% of the laser intensity, >80% of the maximal response amplitude was induced in the PV-INs (Figure 4C). Repetitive laser stimulation revealed that the PV-IN can reliably follow bursts (at 10 Hz) of short (1 ms) laser pulses with the generation of a single action potential (Figure 4D).

Next we characterized the IPSCs recorded in L2/3 PyrN upon laser stimulation. To analyze the STP of the isolated GABAergic synapse between PV-IN and PyrN, we applied a burst of 10 light pulses (1 ms duration) at a frequency of 10 Hz, while recording under voltage-clamp conditions from L2/3 PyrN (Figure 4E). Burst stimulation led to a transient decrease in the amplitude of the optogenetically induced IPSCs in L2/3 PyrN (Figure 4F). The IPSC amplitude was significantly (*p* < 0.05, Wilcoxon paired test) reduced by 40.2% ± 3.4% (*n* = 10 cells, *n* = 6 animals) for a pulse given 0.3 ms after the last stimulus of the burst, by 34.9% ± 2.3% (*n* = 10 cells, *n* = 6 animals) for 0.8 s latency, by 23.1% ± 2.9% (*n* = 10 cells, *n* = 6 animals) for 1.8 latency, and by 15.7% ± 2.4% (*n* = 10 cells, *n* = 6 animals) for 3.8 s latency (Figure 4F; Table 1).

In summary, these experiments revealed prominent STD at the GABAergic synapse between L2/3 IN and L2/3 PyrN, suggesting that a part of STD at GABAergic synapses in L2/3 PyrN observed upon electrical L4 stimulation was mediated by plasticity of GABAergic synapses.

### 3.5. Synaptically induced depolarization of layer 2/3 pyramidal neurons lacks a short-term depression due to balanced plasticity in the glutamatergic and GABAergic system

In order to investigate the overall functional implication of the STD observed at investigated synapses projecting to L2/3 neurons, we next investigated the effect of the electrical burst stimulation on membrane potential responses in L2/3 PyrN. In these experiments we quantified the amplitude of EPSPs in L2/3 PyrN upon different stimulation paradigms, since the amplitude of the EPSPs determines whether the synaptic inputs can be conveyed by action potentials to the next neurons.

**Figure 5:**
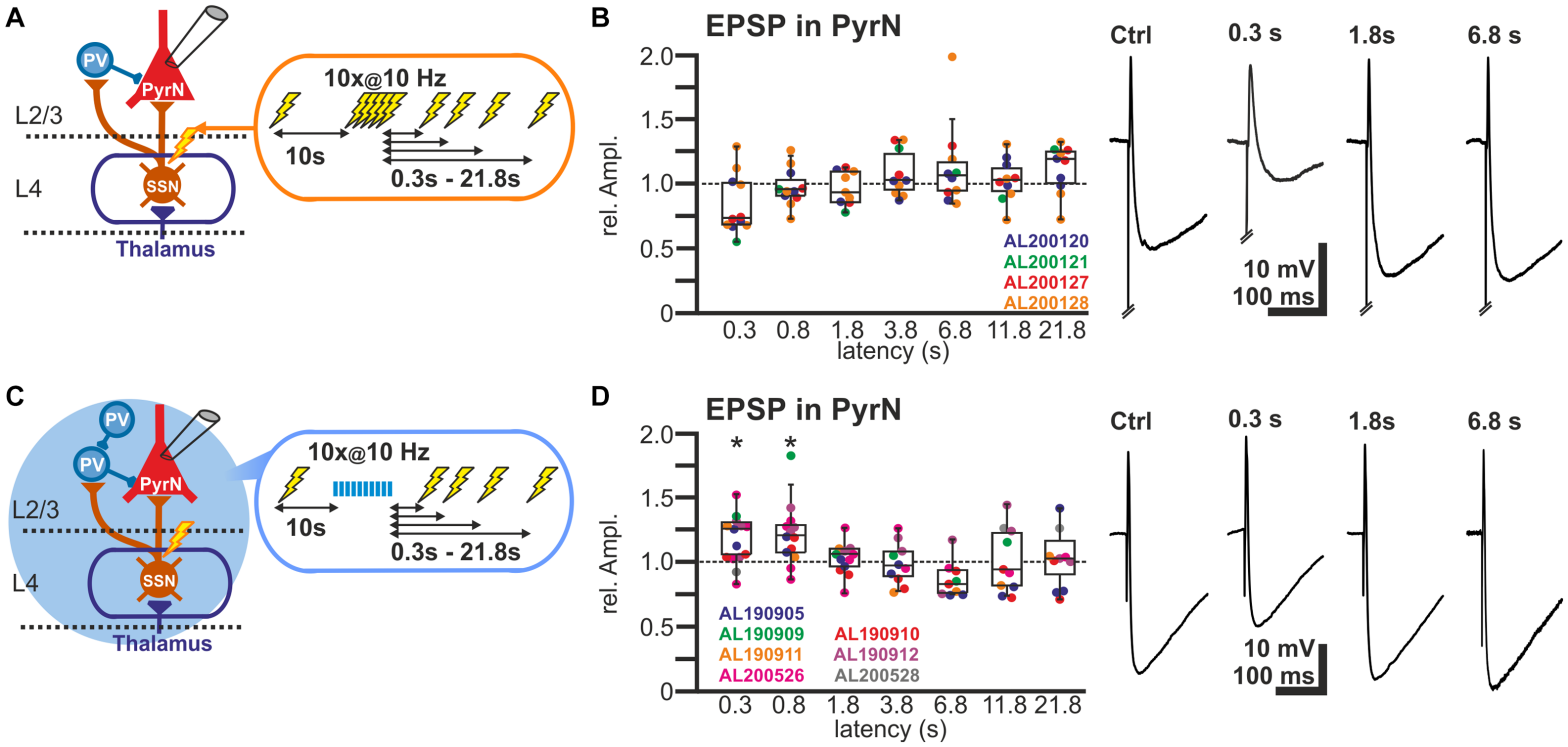
Short-term plasticity of stimulus-induced EPSPs in L2/3 pyramidal neurons. **(A)** Schematic representation of recording and stimulation condition used for the experiment shown in panel **(B)**. Each stimulation protocol consists of a control stimulus, followed by a burst stimulation and test stimuli applied between 0.3 and 21.8 s after the burst. **(B)** Boxplot illustrating the relative EPSP amplitudes, as compared to the control EPSPs, at different latencies after the burst. No significant alterations in the amplitudes were observed. In the right panels, typical voltage traces are displayed. Note that the initial short EPSP was followed by long-lasting hyperpolarization. **(C)** Schematic representation of recording and stimulation condition used for the experiment shown in panel **(D)**. Each stimulation protocol consists of an electrical control stimulus, followed by an optogenetically induced burst stimulation and subsequent electrical test stimulus applied between 0.3 and 21.8 s after the burst. **(D)** Relative amplitude of the EPSPs at different latencies after the optogenetically induced burst. Note the significant increase in the EPSP amplitude 0.3 and 0.8 s after the burst. In the right panels typical current traces are displayed. Note the increased amplitude of EPSPs at latencies of 0.3 and 0.8 s after the burst and the decreased hyperpolarization 0.3 s after the burst. Significance in panel **(B,D)** is indicated by *p < 0.05.

For these experiments we induced, via a monopolar electrode located in L4 an electrical control stimulus, followed by a burst of 10 electrical pulses at a frequency of 10 Hz to induce plasticity and a subsequent test stimulus provided at latencies between 0.3 and 21.8 s (Figure 5A). The experiments demonstrated that the amplitudes of the EPSPs after the burst stimulation were not significantly different from the control stimulus (Figure 5B). The relative EPSP amplitudes, normalized to control EPSP amplitudes, amounted to 84.0% ± 7.0% (*n* = 11 cells, *n* = 4 animals) for a pulse given 0.3 s after the last stimulus of the burst, to 97.8% ± 4.5% (*n* = 11 cells, *n* = 4 animals) for 0.8 s latency, to 96.6% ± 3.9% (*n* = 10 cells, *n* = 4 animals) for 1.8 latency, and to by 108.3% ± 5.6% (*n* = 10 cells, *n* = 4 animals) for 3.8 s latency (Figure 5B; Table 1). On the other hand, the stimulus-induced hyperpolarization was considerably reduced after the burst. In summary, these results indicate that the synaptically-induced depolarization lacks a clear STD after a 10 Hz burst, suggesting that the simultaneous STD of GABAergic and glutamatergic inputs supports reliable signal transmission between L4 and L2/3 PyrN under physiologically relevant conditions.

In order to support this suggestion, we next applied optogenetic burst stimulation, to induce an isolated STD in the GABAergic inputs to L2/3 PyrN. Subsequently we quantified the effect of this procedure by comparing the amplitudes of electrically-induced EPSPs delivered before and at defined latencies after the optogenetic burst (Figure 5C). These experiments revealed that the electrically-evoked EPSP amplitude was significantly augmented after induction of a GABAergic STD by the optogenetic burst (Figure 5D). The EPSP amplitude was significantly increased by 17.4% ± 5.1% (*n* = 14 cells, *n* = 7 animals, *p* = 0.023) for a pulse given 0.3 s after the burst, and by 21.9% ± 6.8% (*n* = 13 cells, *n* = 5 animals, *p* = 0.027) at 0.8 s latency. For pulses given at longer latencies after the burst no significant alterations in EPSP amplitude were observed (Figure 5D; Table 1). At a latency of 0.3 s after the optogenetic burst the membrane was still slightly hyperpolarized by 1.3 ± 0.4 mV (*n* = 14 cells, *n* = 7 animals, *p* = 0.0072), while already at 0.8 s no significant hyperpolarization was found (0.2 ± 0.3 mV, *p* = 0.1378), reflecting the rapid decay of the membrane hyperpolarization (*τ* = 109.2 ± 12 ms). No significant (*r*^2^ = 0.0871) correlation between the small residual hyperpolarization at 0.3 s and the EPSP amplitude was found. Please note the considerable reduction in the stimulus-induced hyperpolarization that reflects the optogenetically induced STD of the GABAergic inputs.

In summary, these results suggest that the simultaneous STD occurring at the GABAergic and glutamatergic synapses in L2/3 supports stable transmission after the 10 Hz burst.

## 4. Discussion

STP is an essential functional element to adapt neuronal circuits to continuous or recurring sensory stimuli (Wark et al., 2007; Rosenbaum et al., 2012). Several *in vitro* and *in vivo* studies investigated in the somatosensory cortex STD during repetitive electrical stimulation of synaptic inputs or sensory stimulation (Reyes et al., 1998; Feldmeyer et al., 2002; Beierlein et al., 2003; Gabernet et al., 2005; Katz et al., 2006; Silberberg and Markram, 2007; Ganmor et al., 2010). While in four studies the recovery of STD was addressed (Chen et al., 2002; Chung et al., 2002; Cohen-Kashi Malina et al., 2013; Li and Glickfeld, 2023), no information on the decay kinetics of STD at EPSCs and IPSCs in L2/3 of the barrel cortex has been published. In order to analyze in detail how excitatory and inhibitory synapses recover after induction of STD, we investigated in L2/3 of the barrel cortex *in vitro* the kinetics of STD for EPSCs, IPSCs, and EPSPs after application of a 10 Hz burst stimulus in L4. We observed that (i) evoked EPSCs in L2/3 PyrN and L2/3 IN showed ca. 35% STD decaying within ≤3.8 s, (ii) that GABAergic projections from L2/3 PV-IN to L2/3 PyrN showed ca. 40% STD decaying within 12 s, (iii) that disynaptic IPSCs observed in L2/3 PyrN upon L4 stimulation showed a STD of >60% with a decay >12 s, and (iv) that L2/3 PyrN exhibited no depression of EPSPs after a burst stimulation, while a selective STD of the GABAergic projections to L2/3 PyrN transiently augmented EPSPs. We conclude from these results that a balanced STD of glutamatergic and GABAergic synapses underlie the stable stimulus-evoked EPSPs in L2/3 PyrN after repetitive stimulation.

### 4.1. Properties of the observed postsynaptic events in L2/3 neurons

In the present study we mainly investigated postsynaptic currents and potentials in L2/3 neurons upon electrical stimulation in L4. The observed EPSC properties are in accordance with previous reports (Helmstaedter et al., 2008; Cohen-Kashi Malina et al., 2013; Pala and Petersen, 2015). On the other hand, we observed that glutamatergic and GABAergic conductance in L2/3 PyrN had comparable amplitudes, in contrast to the dominance of IPSCs in L4 PyrN (Sun et al., 2006), suggesting that feedforward inhibition in L2/3 is weaker as compared to L4.

EPSCs observed in L2/3 PyrN and PV-IN occurred at a latency of ca. 2 ms after the electrical stimulation, in line with a monosynaptic glutamatergic connection (Feldmeyer et al., 2002; Cruikshank et al., 2007; Helmstaedter et al., 2008). IPSCs observed in L2/3 PyrN occurred at a substantially longer latency of 3.3 ms and are to a major extent blocked in the presence of the glutamatergic antagonists CNQX and APV, indicating that mainly excitatory synapses on L2/3 INs projecting to L2/3 PyrN are underlying these disynaptic responses. The observed latency difference of 1.3 ms is in line with the latency difference of 1.5 ms between excitatory and inhibitory inputs found in L4 neurons upon whisker stimulation (Wilent and Contreras, 2005). The shorter latency (2 ± 0.2 ms) and rise time (1.4 ± 0.3 ms) of EPSCs also implies, that the peak amplitude of the EPSC is only marginally affected by the feed forward inhibition, which started (latency 3.3 ± 0.3 ms) and peaked (rise time 4.9 ± 0.8 ms) at significantly later time points. The relatively short latency of the IPSC evoked by electrical stimulation illustrates that GABAergic IN are adapted for rapid signal integration and transmission (Doischer et al., 2008), allowing immediate initiation of action potentials, as demonstrated *in-vivo* upon whisker deflection (Reyes-Puerta et al., 2015a; Vandevelde et al., 2022). In addition, this longer latency of the IPSCs suggests that they originate mainly from GABAergic IN activated by glutamatergic synaptic inputs, and not from directly activated GABAergic inputs. Since we did not observe IPSCs occurring within the latency range of monosynaptic inputs, we assume that direct inhibitory connections from L4 IN or from antidromically activated L4 IN to L2/3 PyrN do not considerably contribute to the inputs under our stimulation conditions.

Whereas the EPSCs rise and decay kinetics of the present study are in line with the properties of a monosynaptic glutamatergic EPSP of the L4 to L2/3 connection (Feldmeyer et al., 2002), the rise time and decay time constants of the IPSCs determined in the present study are considerably longer than reported for unitary IPSP from L2/3 IN to L2/3 PyrN (Hoffmann et al., 2015). The longer rise time for the IPSCs in our study may reflect the jitter of spike induction in the L2/3 IN within this disynaptic response. The longer decay might be due to the stronger GABA release caused by our massive stimulation, which in consequence challenges GABA reuptake that determines IPSC decay kinetics (Farrant and Kaila, 2007).

### 4.2. Properties of the short-term depression in L2/3 synapses

A variety of studies investigated long-term potentiation (Crair and Malenka, 1995; Fox, 2002; An et al., 2012) as well as paired-pulse facilitation/depression in the barrel cortex (Feldmeyer et al., 2002; Beierlein et al., 2003; Helmstaedter et al., 2008; Lefort et al., 2009; Ma et al., 2012; Van Der Bourg et al., 2017). However, as one major function of the whisker to barrel cortex system is the active exploitation of the environment using a series of active whisker movements (Petersen, 2007; Stüttgen and Schwarz, 2018), we ought to characterize STP during and after repetitive synaptic inputs. For this purpose we used a series of inputs provided at 10 Hz, a characteristic frequency of active whisking behavior (Jadhav and Feldman, 2010). Upon an active whisker contact only 0.15 to 0.2 spikes were immediately triggered in each L4 excitatory neuron (Reyes-Puerta et al., 2015b; Yu et al., 2019), while about 0.7 spikes were triggered under this conditions in L2/3 excitatory neurons (Yu et al., 2019). The more reliably spike generation in L2/3 was probably caused by the convergence of multiple synaptic inputs from several L4 spiny stellates to one L2/3 PyrN (Lübke and Feldmeyer, 2007). These *in-vivo* observations indicate that individual L4 to L2/3 synapses probably do not directly follow the active whisking frequency. In contrast, about 2.4 spikes per whisker touch were triggered in L2/3 fast-spiking IN (Yu et al., 2019), suggesting that the disynaptic inhibitory connections investigated can follow the chosen frequency of 10 Hz. In consequence, the amount of STD for EPSCs in L2/3 PyrN is probably overestimated in our study, while the strong STD of IPSCs in PyrN might reliably be obtained under physiological conditions.

For all synapses investigated, we found a considerable STD during such a repetitive stimulation. These observations are in line with previous reports for other synapses in the barrel cortex (Reyes et al., 1998; Feldmeyer et al., 2002; Beierlein et al., 2003; Gabernet et al., 2005; Kapfer et al., 2007; Silberberg and Markram, 2007; Cohen-Kashi Malina et al., 2013) as well as for other cortical areas (Tamás et al., 1997; Zaitsev and Lewis, 2013). In particular they are also consistent with the decreased cortical responses upon 10 Hz whisker stimulation *in-vivo* (Gabernet et al., 2005; Katz and Lampl, 2021) as well as with the reduction the spike frequency of putative INs and L4 PyrNs (Khatri et al., 2004). Only in two cells we observed an initial short-term facilitation for the second pulse. This dominance of STD is in accordance with the observation that typically interstimulus intervals of <100 ms are required to induce paired-pulse facilitation (Fleidervish and Gutnick, 1995; Chen et al., 2002). The synaptic STD can probably be attributed to a depletion of synaptic vesicles from the readily releasable pool, while a desensitization of glutamate and GABA_A_ receptors may also contribute (Chen et al., 2002; Regehr, 2012).

The amount of STD in L2/3 PyrN was more pronounced for GABAergic inputs than for the glutamatergic inputs, in line with previous *in-vitro* results (Feldmeyer et al., 2002; Beierlein et al., 2003; Sun et al., 2006; Hull et al., 2009). The STD of the L2/3 PV-IN to L2/3 PyrN synapses was in the present manuscript tested by optogenetic stimulation. Several studies indicate that optogenetic stimulation by itself can massively enhance short term depression, either by the Ca^2+^ permeability of the ChR2 or by unidentified effects of the used AAVs (Zhang and Oertner, 2007; Jackman et al., 2014). Therefore, we cannot exclude that the observed STD of IPSCs upon optogenetic stimulation was partially caused by such unspecific effects. On the other hand, the comparable kinetics between electrically and optogenetically evoked IPSCs and the smaller amount of STD in the optogenetic experiments suggest that a major part of STD represents physiological effects. In addition, the altered Ca^2+^ handling in the presynapse can probably not influence the STD lasting for several seconds. In our experiments STD of L2/3 PyrN is smaller than the STD of L4 PyrN upon repetitive whisker stimulation *in-vivo* (Cohen-Kashi Malina et al., 2013), suggesting that the sensory adaptation in L4 is more pronounced than in L2/3. This observation is in line with *in-vivo* observation demonstrating that the STD upon repetitive whisker stimulation *in-vivo* is more pronounced in L4 neurons (Cohen-Kashi Malina et al., 2013), than in L2/3 neurons (Katz and Lampl, 2021), although part of this difference may be caused by a higher stimulation frequency used in the L4 study.

### 4.3. Kinetic properties of the short-term depression in L2/3 synapses

The central aspect of our study was to quantify how fast STD decayed at the different synapses. In the present study the kinetics for the release from STD was for the first time quantified for both EPSCs and IPSCs in L2/3 PyrN. We assume that the release from the STD reflects the slow recovery of the readily releasable vesicle pool as well as the decline of glutamate and GABA_A_ receptor desensitization (Regehr, 2012).

We observed in L2/3 PyrN that the release from STD is considerably faster for EPSCs than for IPSCs. The depression of the disynaptic IPSCs in these neurons can originate from a depression of excitatory synapses on GABAergic PV-IN or from a depression of GABAergic synapses on the L2/3 PyrN. However, the EPSCs in identified PV-IN were depressed to a similar amount and with comparable kinetics as EPSCs on L2/3 PyrN, whereas optogenetically-induced IPSCs in L2/3 PyrN showed a substantially longer STD. Therefore, we propose that the slow decay of the IPSCs in L2/3 PyrN was mainly caused by the properties of the GABAergic synapses. Our *in-vitro* results are roughly comparable to *in-vivo* observation in unclassified L2/3 or L4 neurons, where the STD decays within ca. 10 s (Chung et al., 2002). In contrast, a considerable faster decay of the STD was observed in VPM (thalamic ventral posteromedial nucleus) neurons (Chung et al., 2002), at retinogeniculate synapses in the LGN (Chen et al., 2002), in Py2/3 neurons of the visual cortex (Li and Glickfeld, 2023), and in L4 PyrN of the barrel cortex (Cohen-Kashi Malina et al., 2013). Although we cannot exclude that the recording conditions in the present study contribute to the slower STD decay as compared to VPM neurons and L4 PyrN, it’s intriguing to speculate that the longer-lasting STD of L2/3 neurons is an adaptation to the associative functions of this layer (Ayaz et al., 2019), whereas VPN and L4 neurons mainly relay information and discriminate acute features of sensory inputs (Feldmeyer et al., 2013; Harris and Shepherd, 2015). Further studies addressing the recovery rates upon STD for defined neuronal connections are in any way required to resolve whether individual connection show typical kinetics of STP decay.

The stronger STD of IPSCs as well as the slower recovery of IPSCs from STD suggests that a transient phase of reduced feedforward inhibition should appear in a limited interval after the burst stimulation. However, in the present study we did not observe a significant increase in EPSP amplitudes after the end of the burst stimulation. This is in contrast to an *in-vivo* study, reporting such a transient phase of postburst facilitation in about 30% of L4 neurons (Cohen-Kashi Malina et al., 2013). We cannot exclude that the high level of stimulation, which exceeds the physiological response pattern of L4 neurons to whisker stimulation (see section 4.2), exaggerated the synaptic depression at glutamatergic synapses in L2/3 PyrN. Thereby STD of EPSCs might be artificially enhanced and consequently any postburst facilitation will be attenuated. However, the difference may also be caused by different kinetics of STD at excitatory and/or inhibitory synapses between the thalamocortical inputs on L4 neurons investigated by Cohen-Kashi Malina et al. (2013) and the L4 to L2/3 connections investigated in the present study. In addition, the facilitation of neuronal responses in the VPN following repetitive, strong whisker stimulation (Ganmor et al., 2010) may also contribute to this postburst facilitation. In the visual cortex it has been demonstrated that the STD of L2/3 spiking rates can be attributed to a large extent to the depression of L4 to L2/3 synapses, while L4 neurons showed a substantial smaller STD (Li and Glickfeld, 2023). These discrepant observations again emphasize that further studies addressing the recovery rates of STD at precisely defined neuronal connections are compulsory.

### 4.4. Influence of the STD of EPSCs and IPSCs on sensory integration

To keep a neuronal system functional, modifications in the excitatory and inhibitory system must be largely balanced (House et al., 2011; Turrigiano, 2011; Chiu et al., 2019). The stable EPSP amplitudes observed in the present study after the burst stimulation may indicate that in L2/3 the STD of GABAergic IPSCs and glutamatergic EPSCs are balanced under our stimulation condition. The larger STD of IPSCs may be required to adapt the dominance of GABAergic inhibition for the excitation/inhibition ratio at L2/3 inputs in the barrel cortex (Zhang et al., 2011). By such a coherent adaptation of GABAergic and glutamatergic synapses, reliable propagation of sensory information throughout a barrel column can be maintained even when a substantial adaptation of glutamatergic synapses in the relay pathways occurred (Baruchin et al., 2022). On the other hand, several studies demonstrated that STD significantly alters the processing of whisker stimulation in the barrel cortex, suggesting that STP is an important element to adapt cortical information processing *in-vivo* (Khatri et al., 2004; Gabernet et al., 2005; Katz et al., 2006; Cohen-Kashi Malina et al., 2013; Katz and Lampl, 2021).

However, even when the EPSP amplitude was unaffected in L2/3 neuron by a balanced STP of inhibitory and excitatory inputs, the different adaptations of EPSCs and IPSCs might still considerably influence information processing and propagation in the barrel cortex. The prominent adaption of the GABAergic-IN in L2/3 may boost postsynaptic responses upon synaptic inputs from neighboring columns and/or cortical areas (Tremblay et al., 2016; Katz and Lampl, 2021; Staiger and Petersen, 2021), thereby promoting integration of complex sensory stimuli across barrels. Indeed, *in-vivo* experiments demonstrated in the barrel cortex that optogenetic inhibition of PV-IN not only increases the neuronal excitability, but extends the lateral coverage of single whisker stimulation-induced cortical responses (Yang et al., 2017; Yeganeh et al., 2022). In addition, it has been shown that the reduced IPSCs upon repetitive stimulation widens the temporal window for signal integration and, accordingly, decreases the precision of spike generation in L4 PyrN (Gabernet et al., 2005). In line with this, the jitter in spike onset is getting larger in L4 regular spiking units, with repetitive thalamocortical stimulation (Gabernet et al., 2005), indicating that a dominant STD of IPSCs impairs information processing beyond a pure effect on the gain. Finally, simultaneous depression of inhibitory and excitatory synaptic inputs caused a prominent phase shift in spike initiation upon sinusoidal whisker stimulations (Khatri et al., 2004), suggesting a considerable alteration in sensory information processing in the barrel cortex.

Our analysis of IPSCs could not discriminate how different subpopulations of GABAergic IN contribute to these synaptic inputs. PV-INs comprise not only basket cells, but also chandelier neurons (Ascoli et al., 2008). Chandelier-IN in L 2/3 receive excitatory inputs from L4 and L5 (Xu and Callaway, 2009) and project mainly to L2/3 (Helmstaedter et al., 2009b), suggesting that they probably contribute to the observed IPSCs. Somatostatin^+^-interneurons (SOM-INs) target mainly dendrites in the same layer (Riedemann, 2019), and L2/3 SOM-IN receive their inputs mainly from adjacent PyrN (Yu et al., 2019). Thus we cannot exclude that inputs from SOM-IN may contribute to the recorded disynaptic IPSCs. On the other hand, the excitatory inputs to SOM-IN are characterized by prominent facilitation (Silberberg and Markram, 2007; Pala and Petersen, 2015), which is in contrast to the prominent depression observed in the present study and suggests a minor contribution of GABAergic inputs from SOM-IN to the IPSCs. The major subpopulation of GABAergic INs in L2/3 are vasoactive intestinal peptide expressing interneurons (VIP-INs) (Xu et al., 2010). VIP-INs are projecting preferentially to SOM-IN, thereby mediating a disinhibition (Lee et al., 2013; Guet-McCreight et al., 2020). Due to assumed latencies in this trisynaptic circuit they cannot considerably influence the fast monosynaptic EPSCs or EPSPs investigated in this study. On the other hand, their activation might enhance the amplitude of the IPSCs. The distinct STP at inputs to PV-an SOM-INs can cause a shift from a more somatic to a more dendritic inhibition and thus substantially alter neuronal information processing (Seay et al., 2020).

In summary, the observation of STD with distinct decay kinetics at all investigated synapses within L2/3 of the barrel cortex suggests that sensory inputs with complex spatiotemporal properties can induce a rather dynamic pattern of neuronal activity in L2/3. Such distinct STD dynamics within the neuronal circuits may on one hand boost the identification of complex structures hidden in the sensory inputs, like, e.g., texture information. On the other hand, these dynamic properties of sensory inputs can also attenuate the information content about redundant sensory information present in the neocortex.

### 4.5. Suitability of the used methods

In the present study we used in the majority of experiments an electrical stimulation within L4. While we assume that this stimulation paradigm activated axonal connections originating from L4 neurons (Schubert et al., 2007; Feldmeyer et al., 2013), we cannot exclude that also other projections terminating in supragranular layers are stimulated (Feldmeyer et al., 2013; Staiger and Petersen, 2021; Luhmann, 2023). These connections can include few synapses from lemniscal pathways to L3 PyrN (Petreanu et al., 2009; Feldmeyer et al., 2013), but for the somatosensory cortex in particular paralemniscal pathways projecting to supragranular targets are relevant (Feldmeyer et al., 2013; Williams and Holtmaat, 2019; Staiger and Petersen, 2021). However, the paralemniscal pathways make only few (Bureau et al., 2006; Oberlaender et al., 2012; Jouhanneau et al., 2014) and weak (Audette et al., 2018) synapses on the majority of L2/3 neurons, suggesting that EPSC in L2/3 neurons are dominated by inputs from the canonical circuit underlying the lemniscal pathway. We can also not exclude that paralemniscal terminals on L2/3 PV-IN contribute to the EPSCs in these neurons (Audette et al., 2018). Finally, the electrical stimulation can also generate antidromic spikes. But as we did not record antidromic spikes in L2/3 PyrN under our stimulation conditions and did not observe monosynaptic IPSCs upon electrical stimulation, we can exclude that such antidromic stimulation of PyrN and GABAergic IN can contribute to the observed STP, respectively. Due to the prominent GABAergic interconnections between L2/3 IN (Xu and Callaway, 2009), both, electrical stimulation as well as the optogenetic activation of PV-IN will mediate a feedback inhibition of GABAergic INs. However, as this inhibition will occur delayed to the initial synaptic output of the INs, it interferes only marginally with the determination of EPSC and EPSP amplitude. The properties of already evoked IPSCs in L2/3 PyrN will not be affected by a subsequent inhibition of INs. In addition, our stimulation paradigm obviously activates many synaptic terminals on the recorded neurons simultaneously. The observed PSCs and their STP thus reflects averaged properties of all synapses contributing to the PSC, thereby obscuring the possibility that specific connections may show rather diverse properties. Regarding the plasticity of the optogenetically induced IPSCs in L2/3 PyrN, it should be considered that ChR2 has a considerable Ca^2+^ conductance (Nagel et al., 2003), thereby directly influencing the presynaptic plasticity (Jackman et al., 2014). While we thus cannot exclude that the ChR2-induced Ca^2+^ influx enhanced the STD, the fact that electrically induced GABAergic STD is even larger than the optogenetically induced STD indicates that qualitative predictions can be drawn from our observations.

In summary, these considerations suggest that, despite these limitations, the experiments in the present study reveal important information on the STP of the glutamatergic and GABAergic connectivity between L4 and L2/3 neurons. Thus the conclusion drawn from these experiments can contribute to comprehend the dynamic properties of the canonical circuit underlying major aspects of tactile discrimination in the somatosensory cortex.

## Data availability statement

The raw data supporting the conclusions of this article will be made available by the authors, without undue reservation.

## Ethics statement

The animal study was approved by Landesuntersuchungsamt Rheinland-Pfalz, Koblenz, Germany. The study was conducted in accordance with the local legislation and institutional requirements.

## Author contributions

AL: Formal Analysis, Investigation, Writing – review & editing. QW: Formal Analysis, Investigation, Writing – review & editing. MS: Conceptualization, Writing – review & editing. TM: Conceptualization, Funding acquisition, Resources, Supervision, Writing – review & editing. HL: Conceptualization, Funding acquisition, Resources, Supervision, Writing – review & editing. WK: Conceptualization, Formal Analysis, Supervision, Writing – original draft, Writing – review & editing.

## Funding

This research was funded by grants of the Deutsche Forschungsgemeinschaft to HL (DFG LU 375/15-1), to MS (DFG STU 544/3-1), and to TM (CRC 1080, C02).

## Conflict of interest

The authors declare that the research was conducted in the absence of any commercial or financial relationships that could be construed as a potential conflict of interest.

## Publisher’s note

All claims expressed in this article are solely those of the authors and do not necessarily represent those of their affiliated organizations, or those of the publisher, the editors and the reviewers. Any product that may be evaluated in this article, or claim that may be made by its manufacturer, is not guaranteed or endorsed by the publisher.
